# Supplementary material for: Biological functions of the autophagy-related proteins Atg4 and Atg8 in Cryptococcus neoformans
Source: PLoS One. 2020 Apr 6;15(4):e0230981. doi: 10.1371/journal.pone.0230981 (PMC7135279; doi:10.1371/journal.pone.0230981)
Supplement: S1 Raw images — (PDF) [file pone.0230981.s001.pdf]

S1_Raw_Images. Raw blot and electrophoresis gel images used in this manuscript.

S1_Raw_Figs1-4: Western blot images were visualized using ImageQuant LAS (GE Healthcare) with the following setting conditions: Chemiluminescence Method, Exposure Time defined as Auto and Standard Sensitivity/Resolution.

S1_Raw_Fig5: Electrophoresis gel images was captured using ImageQuant LAS (GE Healthcare) with the following setting conditions: EtBr UV (Trans-illumination) Fluorescence Method, Exposure Time defined as Auto and Standard Sensitivity/Resolution.

S1_Raw_Figs6-7: Southern blot images were visualized using ImageQuant LAS (GE Healthcare) with the following setting conditions: Chemiluminescence Method, Exposure Time defined as Auto and Standard Sensitivity/Resolution.

All captured blot and gel images were exported to TIF format and cropped to be used in the main body and in the supplementary information of this manuscript.

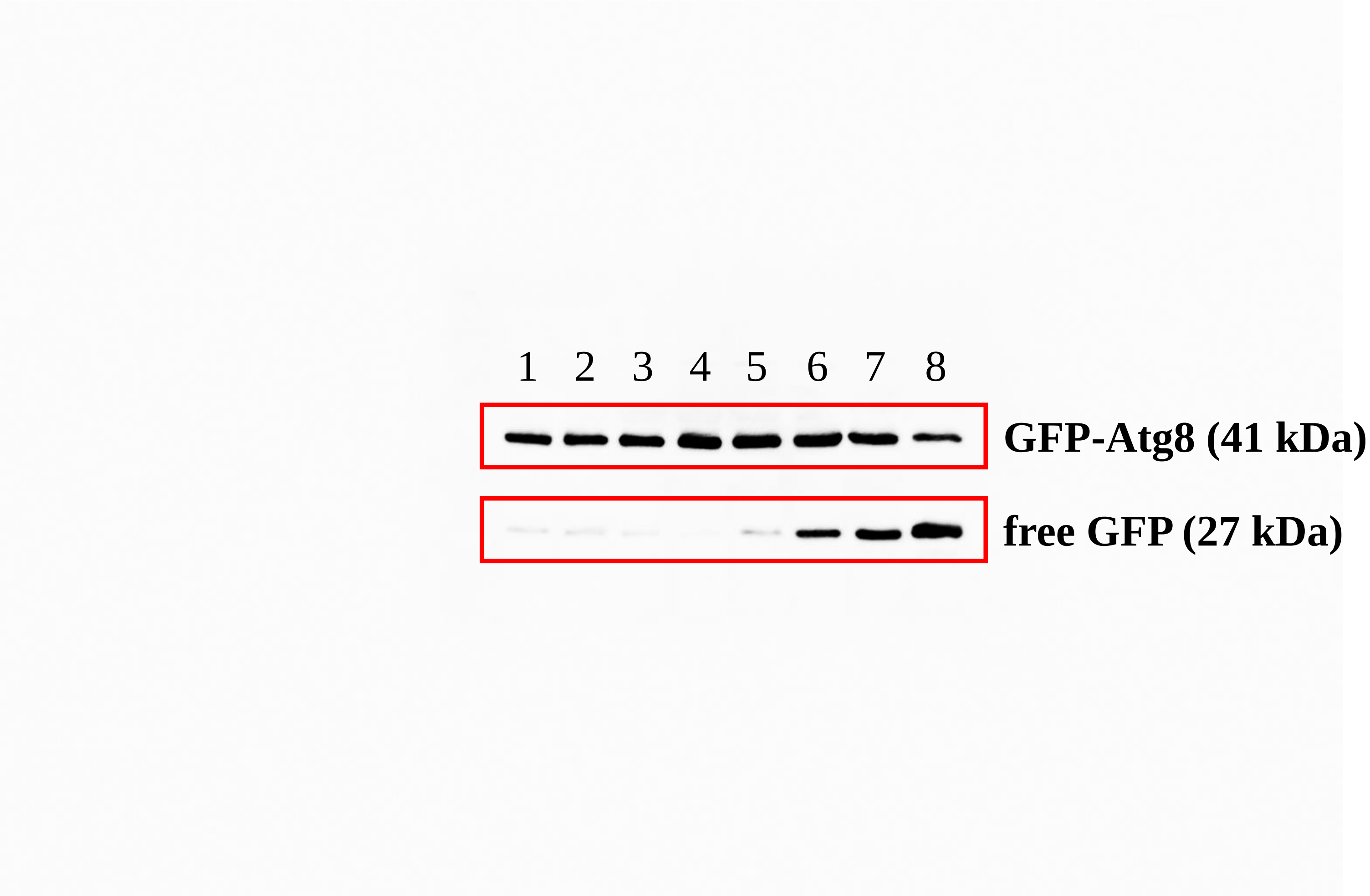

KN99α (anti-GFP):
1: 0h induction in YPD
2: 1h induction in YPD
3: 2h induction in YPD
4: 4h induction in YPD
5: 0h induction in SD/-N/-AA
6: 1h induction in SD/-N/-AA
7: 2h induction in SD/-N/-AA
8: 4h induction in SD/-N/-AA

S1_Raw_Fig1. Raw Western blot image of GFP-Atg8 expressions in KN99α (correspond to Figure 4A top pannel).

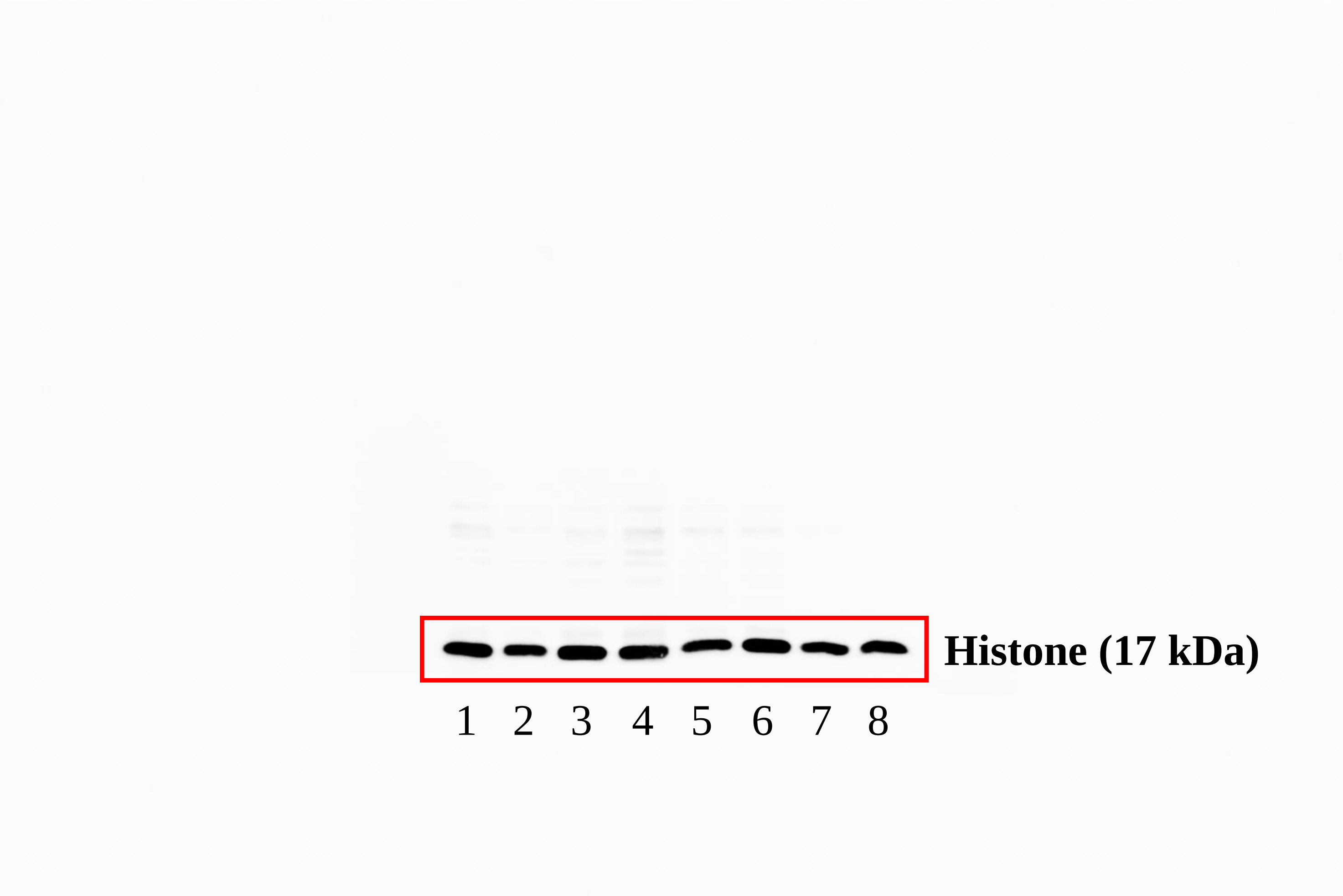

KN99α (anti-histone):
1: 0h induction in YPD
2: 1h induction in YPD
3: 2h induction in YPD
4: 4h induction in YPD
5: 0h induction in SD/-N/-AA
6: 1h induction in SD/-N/-AA
7: 2h induction in SD/-N/-AA
8: 4h induction in SD/-N/-AA

S1_Raw_Fig2. Raw Western blot image of histone expressions in KN99α (correspond to Figure 4A bottom pannel). The S1_Raw_Fig1 membrane was stripped to remove anti-GFP antibody and then incubated with primary rabbit anti-histone.

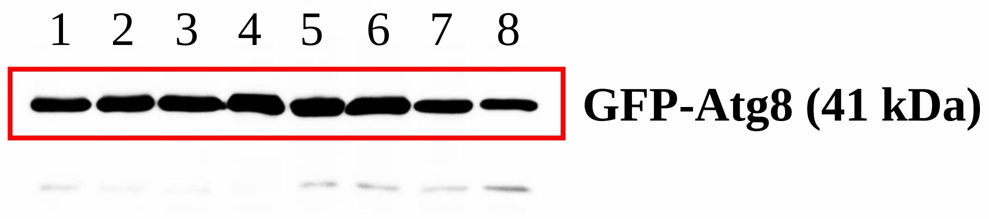

atg4 **(anti-GFP):**
1: 0h induction in YPD
2: 1h induction in YPD
3: 2h induction in YPD
4: 4h induction in YPD
5: 0h induction in SD/-N/-AA
6: 1h induction in SD/-N/-AA
7: 2h induction in SD/-N/-AA
8: 4h induction in SD/-N/-AA

S1_Raw_Fig3. Raw Western blot image of GFP-Atg8 expressions in *atg4* mutant (correspond to Figure 4B top pannel).

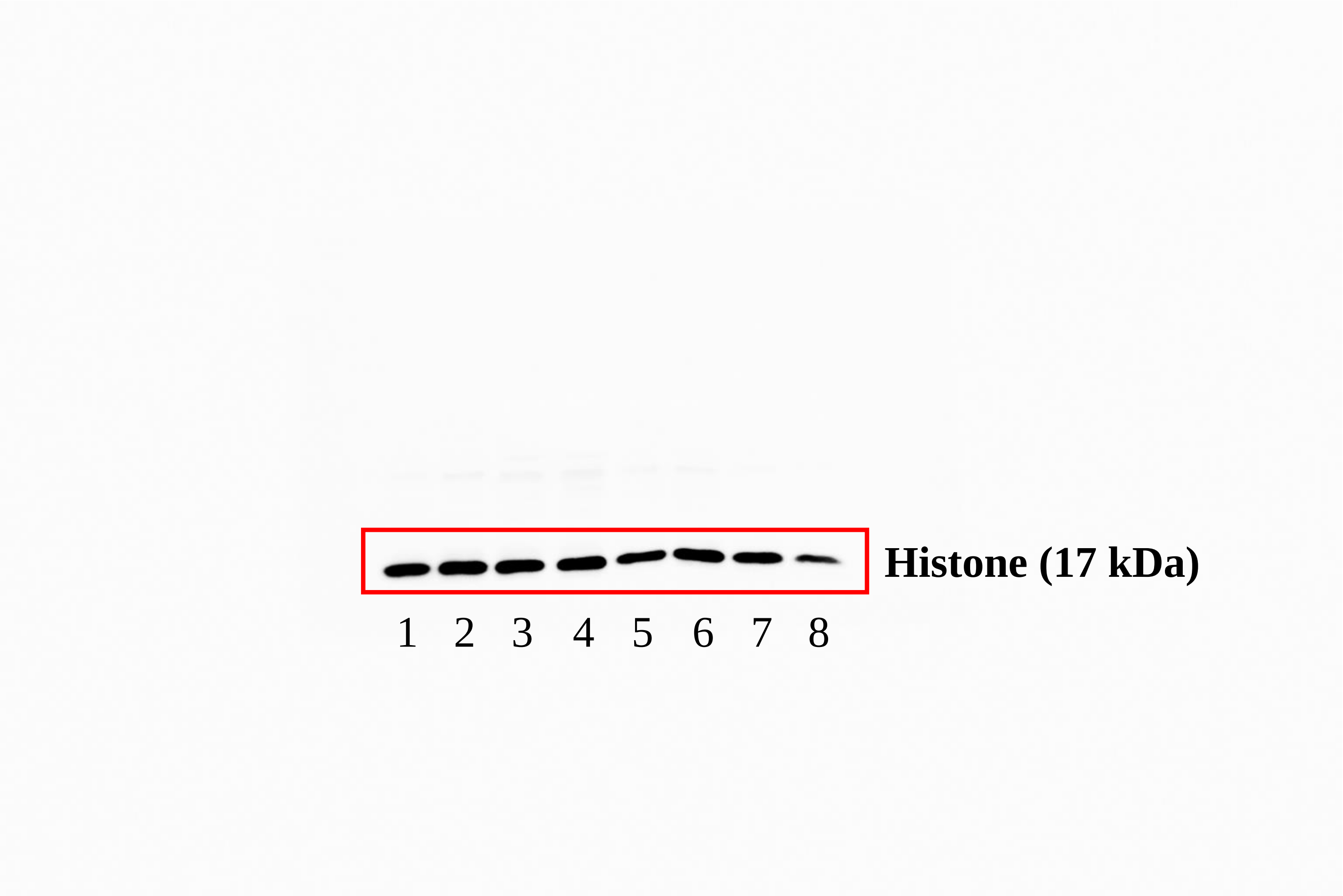

***atg4* (anti-histone):**
1: 0h induction in YPD
2: 1h induction in YPD
3: 2h induction in YPD
4: 4h induction in YPD
5: 0h induction in SD/-N/-AA
6: 1h induction in SD/-N/-AA
7: 2h induction in SD/-N/-AA
8: 4h induction in SD/-N/-AA

S1_Raw_Fig4. Raw Western blot image of histone expressions in *atg4* mutant (correspond to Figure 4B bottom pannel). The S1_Raw_Fig3 membrane was stripped to remove anti-GFP antibody and then incubated with primary rabbit anti-histone.

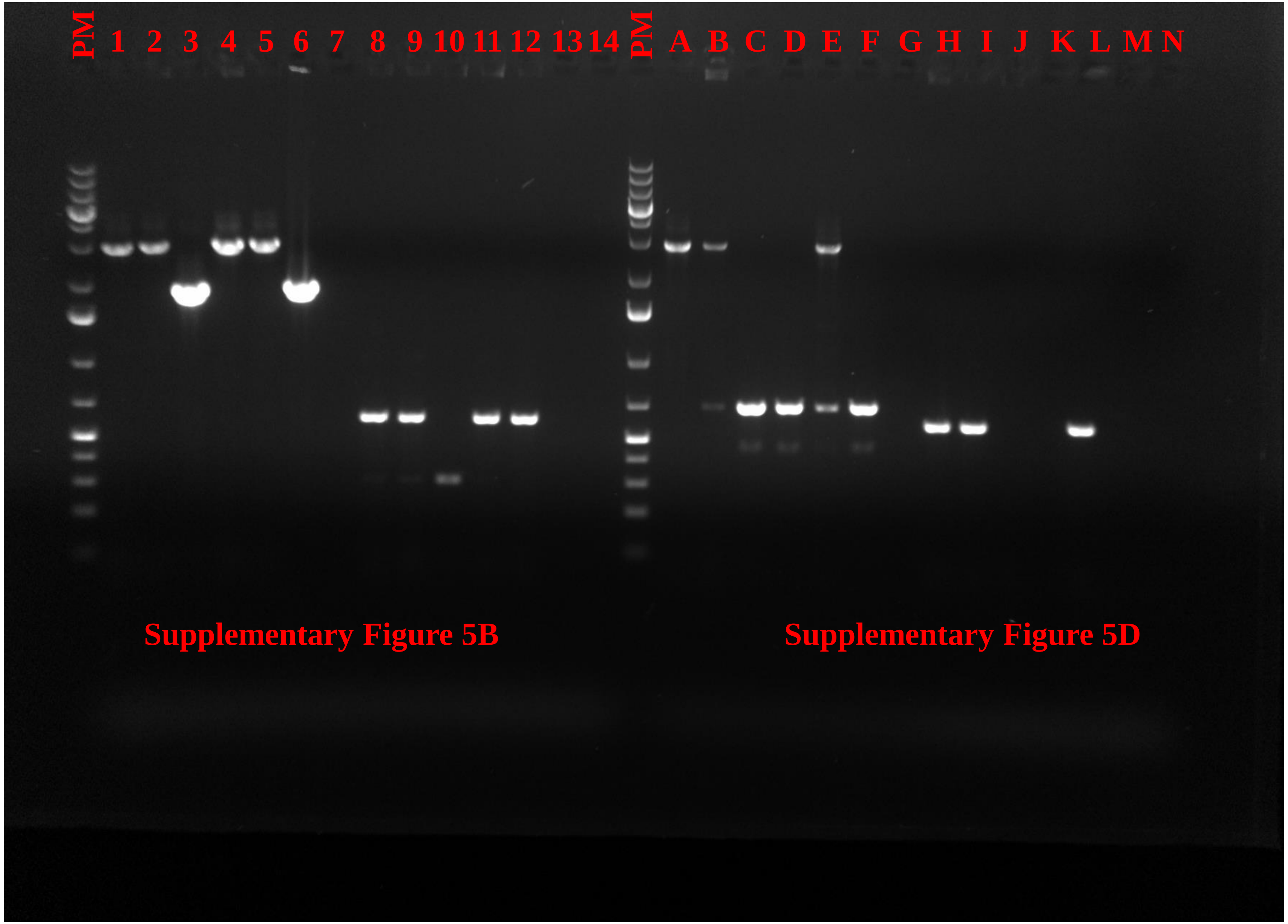

Supplementary Figure 5B

1-7: Confirmatory PCR for *ATG4* deletion diagnosis using the primers: Atg4ScF and Atg4ScR.

8-14: Confirmatory PCR for *ATG4* deletion diagnosis using the primers: Atg4ScF and KanMXR.

Supplementary Figure 5D

A-G: Confirmatory PCR for *ATG8* deletion diagnosis using the primers: Atg8ScF and Atg8ScR.

H-N: Confirmatory PCR for *ATG4* deletion diagnosis using the primers: Atg8ScF and KanMXR.

PM: 1 Kb Plus DNA Ladder (Thermo Fischer Scientific)

S1_Raw_Fig5. Raw electrophoresis gel image of the *ATG4* (YNL223W) and *ATG8* (YBL078C) genes deletion in *S. cerevisiae* (correspond to Supplementary Figures 5B and 5D).

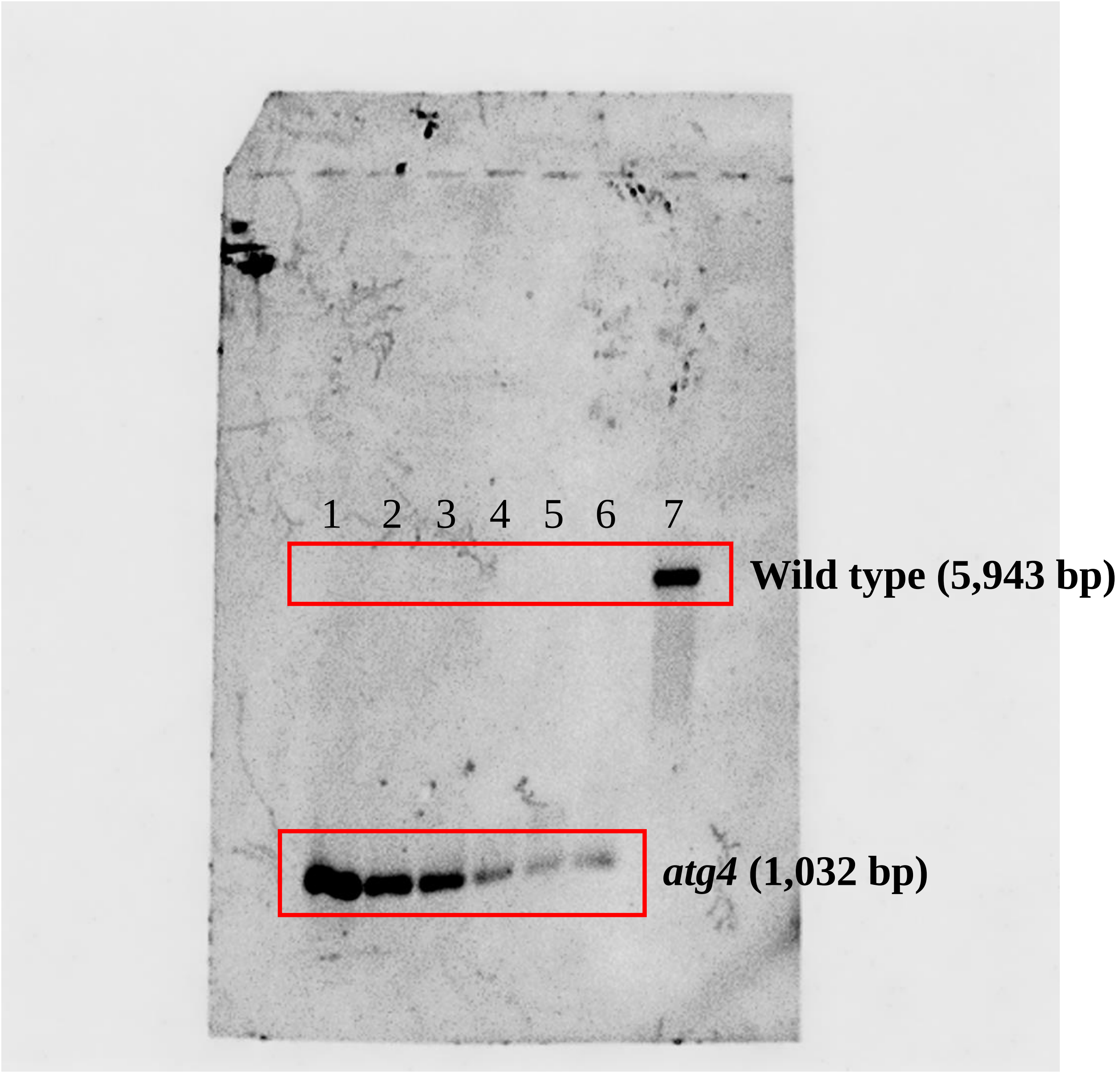

1: colony 1 growing in YPD+Hyg
2: colony 2 growing in YPD+Hyg
3: colony 3 growing in YPD+Hyg
4: colony 4 growing in YPD+Hyg
5: colony 5 growing in YPD+Hyg
6: colony 6 growing in YPD+Hyg
7: KN99α (WT)

S1_Raw_Fig6. Raw Southern blot image of the *ATG4* (CNAG_02662) gene deletion in *C. neoformans* (correspond to Supplementary Figures 6B).

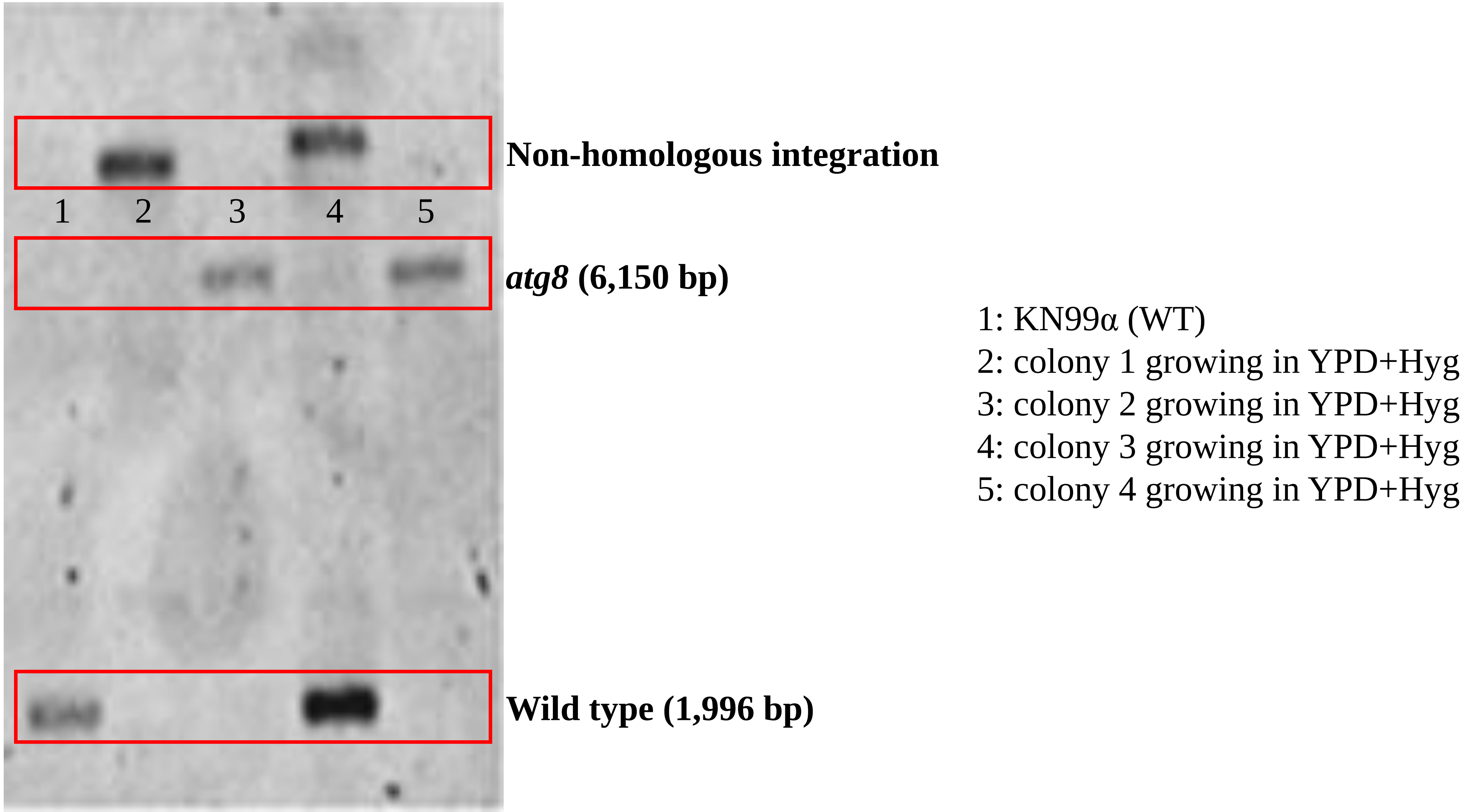

S1_Raw_Fig7. Southern blot image of the *ATG8* (CNAG_00816) gene deletion in *C. neoformans* (correspond to Supplementary Figures 6D). This is not the raw figure, but is the crop made soon after the capture, I retrieved it from an email the student sent. The raw captured image was lost when the computer HD crashed.
