# Supplementary material for: Biological functions of the autophagy-related proteins Atg4 and Atg8 in Cryptococcus neoformans
Source: PLoS One. 2020 Apr 6;15(4):e0230981. doi: 10.1371/journal.pone.0230981 (PMC7135279; doi:10.1371/journal.pone.0230981)
Supplement: S1 Table — (PDF) [file pone.0230981.s002.pdf]

S1 Table. Yeast strains used in this work.

Strain	Microorganism	Genotype	Source
Y2HGold	*S. cerevisiae*	MATa, *trp1-901*, *leu2-3*, *112*, *ura3-52*, *his3-200*, *gal4*Δ, *gal80*Δ, *LYS2-GAL1*$_{UAS}$–*Gal1*$_{TATA}$–*His3*, *GAL2*$_{UAS}$–*Gal2*$_{TATA}$–*Ade2* *URA3-MEL1*$_{UAS}$–*Mel1*$_{TATA}$ *AUR1-C MEL1*	Clontech, Takara Bio
Y2HGold (+)	*S. cerevisiae*	Y2HGold; pGBKT7-53, pGADT7-T	This study
Y2HGold (–)	*S. cerevisiae*	Y2HGold; pGBKT7-Lam, pGADT7-T	This study
pGBKT7-*ATG8* + pGADT7-*ATG3*	*S. cerevisiae*	Y2HGold; pGBKT7-*ATG8*, pGADT7-*ATG3*	This study
pGBKT7-*ATG8* + pGADT7-*ATG4*	*S. cerevisiae*	Y2HGold; pGBKT7-*ATG8*, pGADT7-*ATG4*	This study
pGBKT7-*ATG8* + pGADT7-*ATG7*	*S. cerevisiae*	Y2HGold; pGBKT7-*ATG8*, pGADT7-*ATG7*	This study
BY4741	*S. cerevisiae*	MATa, *his3Δ1*, *leu2Δ0*, *met15Δ0*, *ura3Δ0*	University of Melbourne
atg4Δ	*S. cerevisiae*	BY4741; *atg4*-G418^R	This study
atg4Δ+CnATG4	*S. cerevisiae*	BY4741; *atg4*-G418^R, pYES2-*CnATG4*	This study
atg8Δ	*S. cerevisiae*	BY4741; *atg8*-G418^R	This study
atg8Δ+CnATG8	*S. cerevisiae*	BY4741; *atg8*-G418^R, pYES2-*CnATG8*	This study
KN99α	*C. neoformans*	MATα, wild type	Duke University Medical Center
atg4	*C. neoformans*	KN99α; *atg4*-Hyg^R	This study
atg8	*C. neoformans*	KN99α; *atg8*-Hyg^R	This study
KN99α+GFP-Atg8	*C. neoformans*	KN99α; pCN50-*ATG8*	This study
atg4+GFP-Atg8	*C. neoformans*	KN99α; *atg4*-Hyg^R, pCN50-*ATG8*	This study
