# Supplementary material for: Biological functions of the autophagy-related proteins Atg4 and Atg8 in Cryptococcus neoformans
Source: PLoS One. 2020 Apr 6;15(4):e0230981. doi: 10.1371/journal.pone.0230981 (PMC7135279; doi:10.1371/journal.pone.0230981)
Supplement: S2 Table — (PDF) [file pone.0230981.s003.pdf]

S2 Table. List of oligonucleotides used in this study.

Primer	Direction	Sequence (5' → 3')	Use
MAV279	Sense	CATGGAGGCCGAATTCATGGTCCGAAGCAAGTTTAAG	pGBKT7 cDNA *ATG8*
MAV280	Antisense	GCAGGTCGACGGATCCTTACTCGGAGATGGCGTATTG	pGBKT7 cDNA *ATG8*
MAV435	Sense	GGAGGCCAGTGAATTCATGAATAACCCACTTTTAGCTATC	pGADT7 cDNA *ATG3*
MAV436	Antisense	CGAGCTCGATGGATCCTTAGAGGGCAGTGGACGTAG	pGADT7 cDNA *ATG3*
MAV437	Sense	GGAGGCCAGTGAATTCATGTCATCCCCGGCCTCTAC	pGADT7 cDNA *ATG4*
MAV438	Antisense	CGAGCTCGATGGATCCTTACAACAAATTATCGCCGTTCGG	pGADT7 cDNA *ATG4*
MAV439	Sense	GGAGGCCAGTGAATTCATGGCGCCACTCCAGTTCCAG	pGADT7 cDNA *ATG7*
MAV440	Antisense	CGAGCTCGATGGATCCTCACTCTTCCCCCTCGCTATC	pGADT7 cDNA *ATG7*
Atg4ScF	Sense	ATGCAGAGGTGGCTACAACTGTGGAAAATGGATTTGGTACAAAAAG TGTCCGTACGCTGCAGGTCGAC	BY4741 *atg4Δ*
Atg4ScR	Antisense	CTAGCATTTTTCATCAATAGGACTGTGAATACCTACCGTTTCCTTCTC TAATCGATGAATTCGAGCTCG	BY4741 *atg4Δ*
Atg8ScF	Sense	ATGAAGTCTACATTTAAGTCTGAATATCCATTTGAAAAAAGGAAGG CGGACGTACGCTGCAGGTCGAC	BY4741 *atg8Δ*
Atg8ScR	Antisense	CTACCTGCCAAATGTATTTTCTCCTGAGTAAGTGACATACAAAAACC CGTATCGATGAATTCGAGCTCG	BY4741 *atg8Δ*
KanMXR	Antisense	CAGCACGTGTCTTGTAGTTCC	KanMX6 Diagnostic PCR
Atg4CnF	Sense	GACTCACTATAGGGAATATTACACAATGTCATCCCCGGCCTC	pYES2-*CnATG4*
Atg4CnR	Antisense	TAATTACATGATGCGGCCCTTACAACAAATTATCGCCGTTCG	pYES2-*CnATG4*
Atg8CnF	Sense	GACTCACTATAGGGAATATTACACAATGGTCCGAAGCAAGTT	pYES2-*CnATG8*
Atg8CnR	Antisense	TAATTACATGATGCGGCCCTTACTCGGAGATGGCGTATTG	pYES2-*CnATG8*
MAV412	Sense	CGTAGTATTCGTTCCTGCAG	Double-joint Left arm *atg4*
MAV413	Antisense	CTCCAGCTCACATCCTCGCAGTGCTTAATCTGAAAACACGTC	Double-joint Left arm *atg4*
MAV414	Sense	GAAGCTAGTTTCTACATCTCTTCACTGTATCAACTTCACAAAGGG	Double-joint Right arm *atg4*
MAV415	Antisense	CTGCGTGGATTGGTAAGCTC	Double-joint Right arm *atg4*
MAV416	Sense	GACGTGTTTTCAGATTAAGCACTGCGAGGATGTGAGCTGGAG	Double-joint Left marker *atg4*
PCRP211	Antisense	CCCGAACATCGCCTCGCTC	Double-joint Left marker *atg4*
PRCP212	Sense	ATCCCCATGTGTATCACTGGC	Double-joint Right marker *atg4*
MAV417	Antisense	CCCTTTGTGAAGTTGATACAGTGAAGAGATGTAGAAACTAGCTTC	Double-joint Right marker *atg4*
MAV418	Sense	GTTTATATAGGGCGAGGGGATG	Double-joint Diagnostic PCR *atg4*
MAV162	Antisense	GACTCACCTTGGGCAGTGGG	Double-joint Diagnostic PCR *atg4*
MAV253	Sense	CAGGAGAAGAGCCAACTCGGC	Double-joint Left arm *atg8*
MAV256	Antisense	CTCCAGCTCACATCCTCGCAGGTTGCTACTGAAATAGGTG	Double-joint Left arm *atg8*
MAV257	Sense	GAAGAGATGTAGAAACTAGCTTCCCAGTACGATAATACTGTTGC	Double-joint Right arm *atg8*
MAV254	Antisense	AGAGTATGCGCCTGGTACGCC	Double-joint Right arm *atg8*
MAV255	Sense	CACCTATTTCAGTAGCAACCTGCGAGGATGTGAGCTGGAG	Double-joint Left marker *atg8*
PCRP211	Antisense	CCCGAACATCGCCTCGCTC	Double-joint Left marker *atg8*
PRCP212	Sense	ATCCCCATGTGTATCACTGGC	Double-joint Right marker *atg8*
MAV258	Antisense	GCAACAGTATTATCGTACTGGGAAGCTAGTTTCTACATCTCTTC	Double-joint Right marker *atg8*
MAV277	Sense	TAGAGATGTCTCGGACAAGG	Double-joint Diagnostic PCR *atg8*
MAV162	Antisense	GACTCACCTTGGGCAGTGGG	Double-joint Diagnostic PCR *atg8*
MAV493	Sense	CGAGCTGTACGGATCCATGGTCCGAAGCAAGTTTAAGGATG	pCN50-*ATG8*
MAV504	Antisense	GGCGGCCGTTACTAGTCATATCAAAGATCTGAAGTGGACGG	pCN50-*ATG8*
