# Supplementary figures and images for: Biological functions of the autophagy-related proteins Atg4 and Atg8 in Cryptococcus neoformans
Source: PLoS One. 2020 Apr 6;15(4):e0230981. doi: 10.1371/journal.pone.0230981 (PMC7135279; doi:10.1371/journal.pone.0230981)

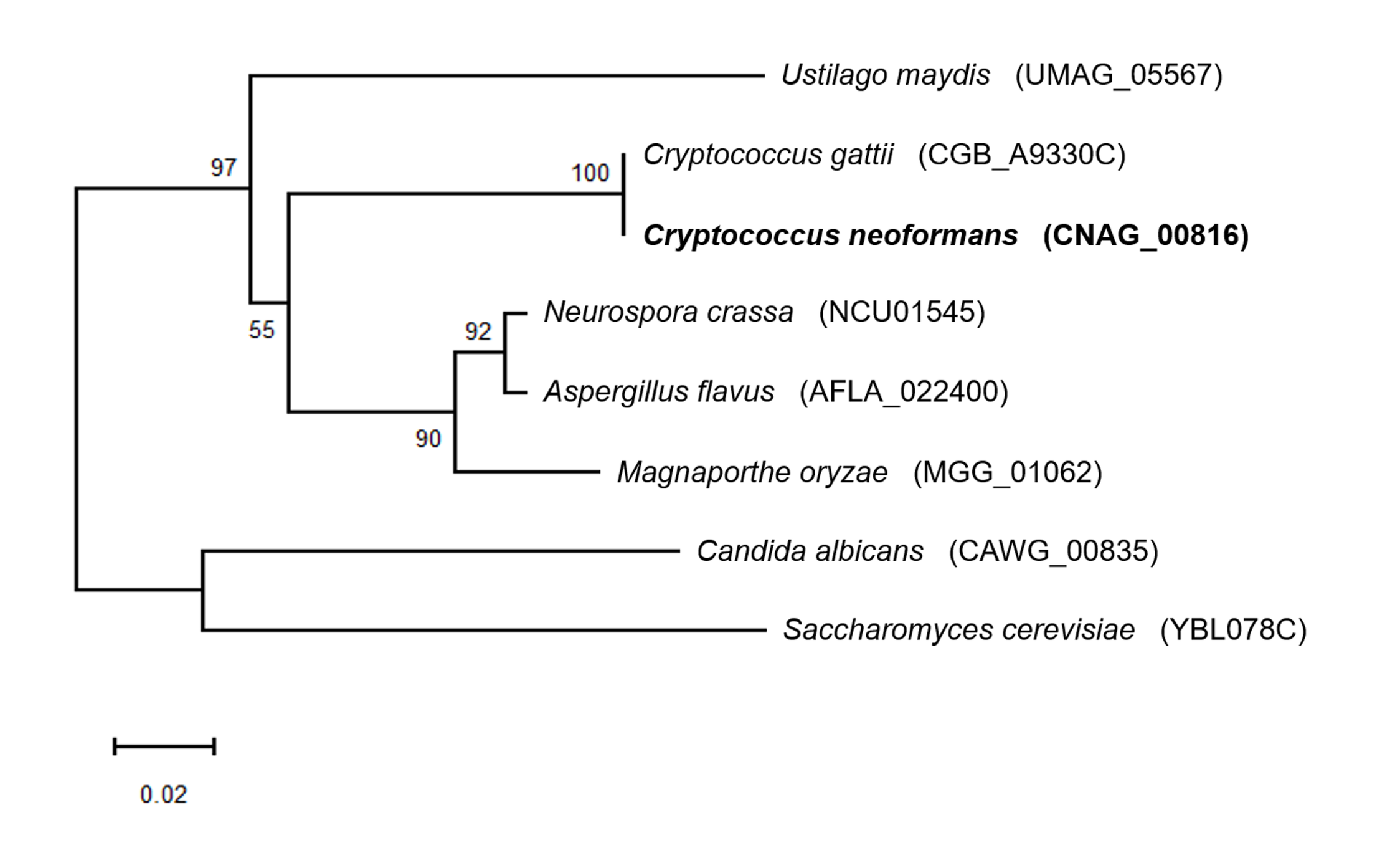

Supplement: S1 Fig — The tree was constructed by the Neighbor-Joining method using the software MEGA v. 10.0.5. The evolutionary distances were computed using the Poisson correction method. The values of the branches indicate the percentages of replicates in which the associated taxa clustered in the bootstrap analysis (1,000 replicates). (TIF) [file pone.0230981.s004.tif]

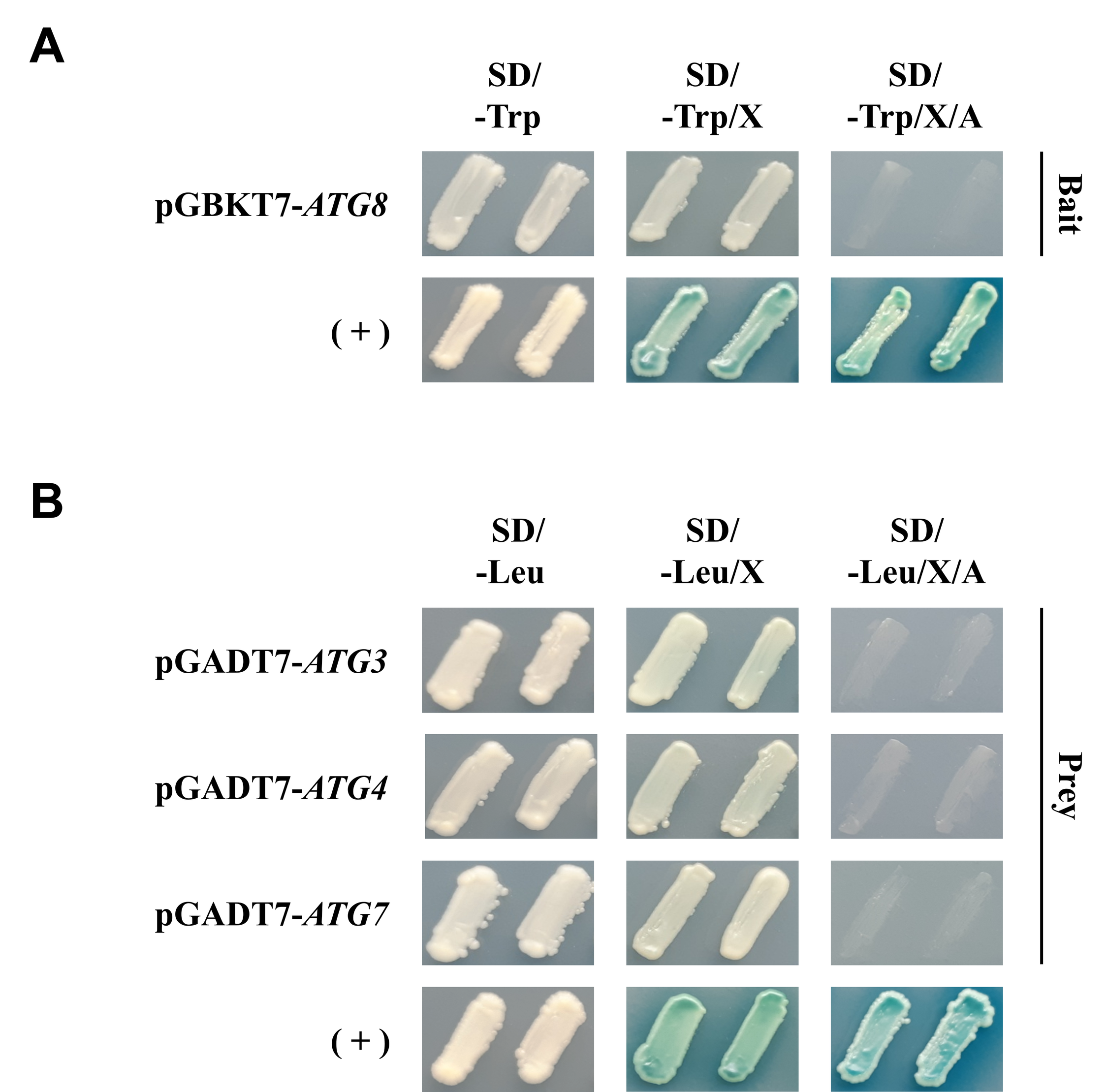

Supplement: S2 Fig — Alignment using the ClustalW program [33]. Identical amino acids in all sequences are indicated in gray. Conserved glycine (G) residues in the C-terminal region are highlighted with a black background. The Ubiquitin-like superfamily domain is indicated by the dashed line. Filled arrows = predicted Atg4 protein binding sites; unfilled arrows = predicted Atg7 protein binding sites. Species and protein accession number: Sc = Saccharomyces cerevisiae (YBL078C); Ca = Candida albicans (CAWG_00835); Cn = Cryptococcus neoformans (CNAG_00816); Cg = Cryptococcus gattii (CGB_A9330C); Nc = Neurospora crassa (NCU01545); Af = Aspergillus flavus (AFLA_022400); Mo = Magnaporthe oryzae (MGG_01062); Um = Ustilago maydis (UMAG_05567). (TIF) [file pone.0230981.s005.tif]

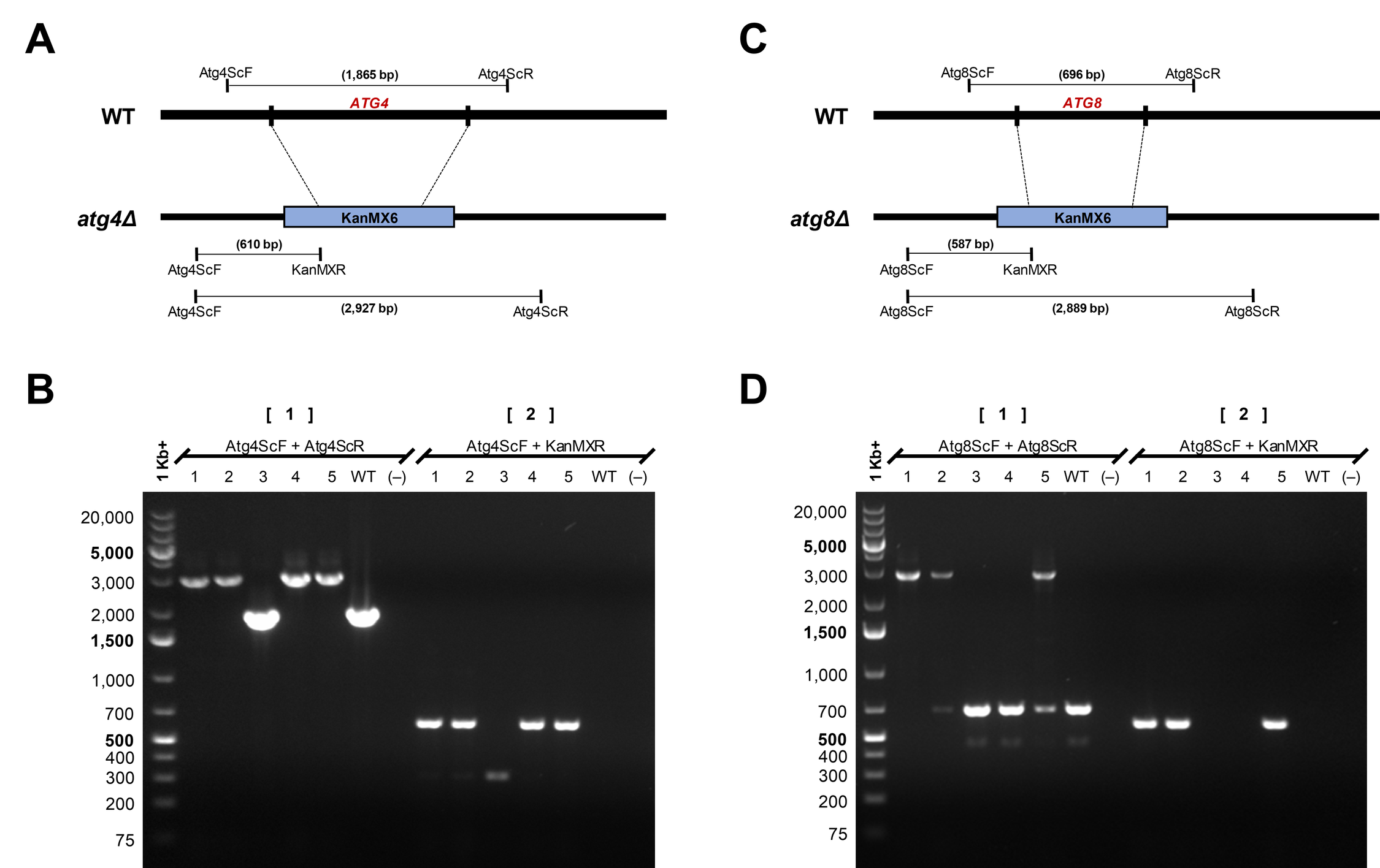

Supplement: S3 Fig — The cloned vectors do not autonomously activate the reporter genes. (A) Growth of Y2HGold yeast strain transformed with the indicated bait (pGBKT7-ATG8). (B) Growth of Y2HGold yeast strain transformed individually with the indicated preys (pGADT7-ATG3/ATG4/ATG7). Two independent clones were tested in a total of n = 2 independent experiments. Plates incubated for 5 days at 30°C. Assay performed with the Matchmaker™ Gold Yeast Two-Hybrid System (Clontech). SD: synthetic dextrose medium; Leu: leucine; Trp: tryptophan; X: 40 μg/mL X-α-Gal; A: 200 ng/mL Aureobasidin A; (+): pair of plasmids pGBKT7-53 and pGADT7-T used as positive control. (TIF) [file pone.0230981.s006.tif]

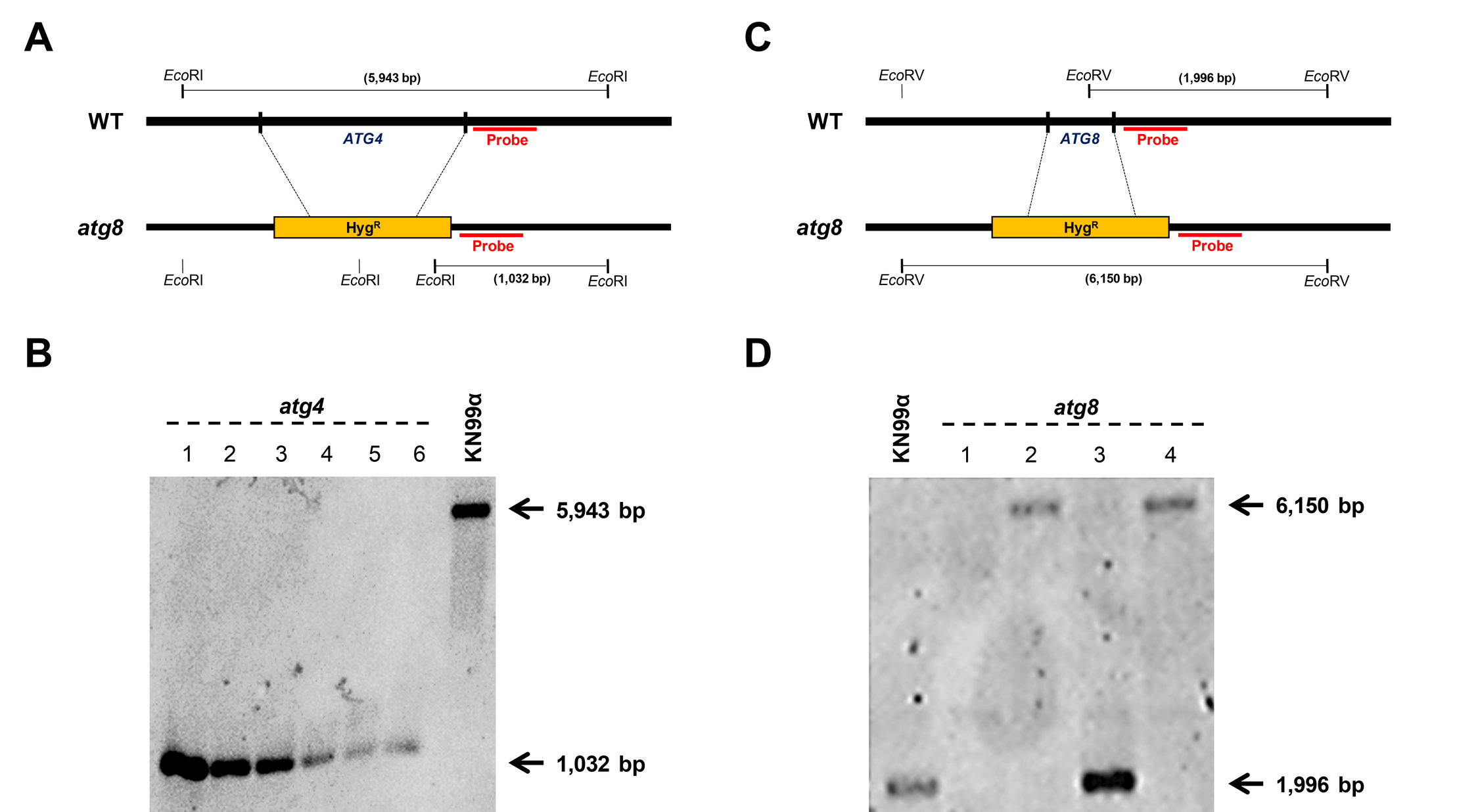

Supplement: S4 Fig — (A) Atg4 from S. cerevisiae (YNL223W); 494 amino acids. (B) Atg4 from C. neoformans (CNAG_02662); 1,185 amino acids. Illustration of the protein domains using the IBS 1.0.3 software (CUCKOO Workgroup). Domains identified using the NCBI platform (Conserved Domain Database) [73]. (C) Multiple alignment of Atg4 homologous amino acid sequences. Region surrounding the active cysteine residue. Identical amino acids in all sequences are highlighted in gray. Catalytic residue of cysteine among the different members of the Atg4 family is indicated in black. Alignment using the ClustalW program [33]. Species and GenBank accession number: Sc = Saccharomyces cerevisiae (YNL223W); Cn = Cryptococcus neoformans (CNAG_02662); Cg = Cryptococcus gattii (CGB_K2500C); Nc = Neurospora crassa (NCU02433); Af = Aspergillus flavus (AFLA_104050); Mo = Magnaporthe oryzae (MGG_03580); Um = Ustilago maydis (UMAG_05142). (TIF) [file pone.0230981.s007.tif]

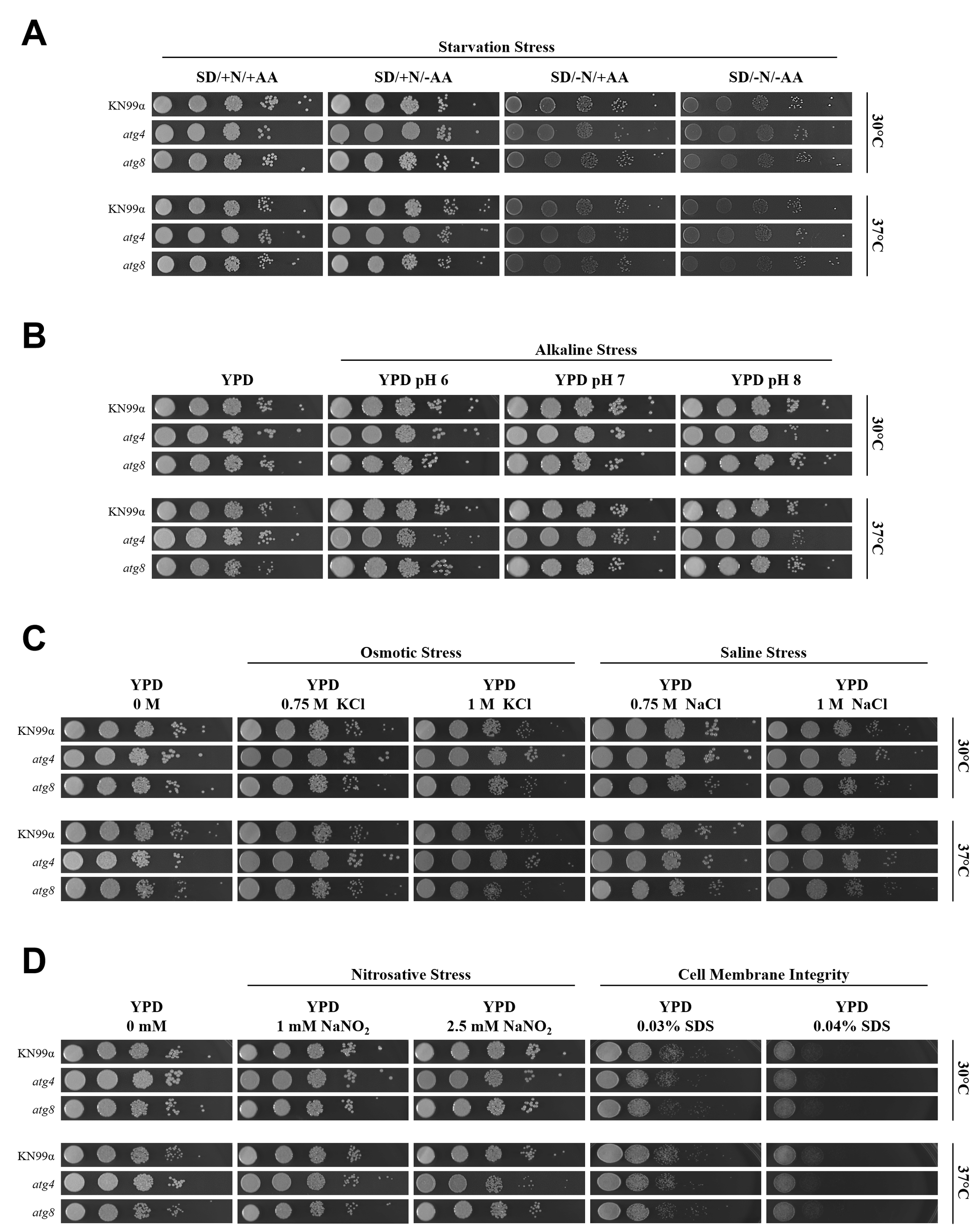

Supplement: S5 Fig — (A) Schematic representation of ATG4 deletion by homologous recombination. (B) Confirmatory deletion performed by diagnostic PCR. Atg4ScF + Atg4ScR: atg4Δ amplicon with 2,927 bp; Atg4ScF + KanMXR: atg4Δ amplicon with 610 bp. Colonies 1, 2, 4 and 5 are atg4Δ. (C) Schematic representation of ATG8 deletion by homologous recombination. (D) Confirmatory deletion performed by diagnostic PCR. Atg8ScF + Atg8ScR: atg8Δ amplicon with 2,889 bp; Atg8ScF + KanMXR: atg8Δ amplicon with 587 bp. Colonies 1, 2 and 5 are atg8Δ. Atg4ScF, Atg4ScR, Atg8ScF, Atg8ScR and KanMXR: primers used in PCR. Lanes 1–5: colonies growing in selective medium. KanMX6: G418 resistance. WT: wild type BY4741 gDNA, (–): negative control. 1 Kb+: GeneRuler 1 Kb Plus DNA Ladder (Thermo Fisher Scientific). (TIF) [file pone.0230981.s008.tif]
